# Formal representation of ambulatory assessment protocols in HTML5 for human readability and computer execution

**DOI:** 10.3758/s13428-018-1148-y

**Published:** 2018-11-07

**Authors:** Nikolaos Batalas, Vassilis-Javed Khan, Minita Franzen, Panos Markopoulos, Marije aan het Rot

**Affiliations:** 1 Department of Industrial Design, Eindhoven University of Technology, Eindhoven, The Netherlands; 2 Department of Psychology, University of Groningen, Groningen, The Netherlands

**Keywords:** Ambulatory assessment, Experience sampling, HTML, Protocol representations, Data collection software systems

## Abstract

Ambulatory assessment (AA) is a research method that aims to collect longitudinal biopsychosocial data in groups of individuals. AA studies are commonly conducted via mobile devices such as smartphones. Researchers tend to communicate their AA protocols to the community in natural language by describing step-by-step procedures operating on a set of materials. However, natural language requires effort to transcribe onto and from the software systems used for data collection, and may be ambiguous, thereby making it harder to reproduce a study. Though AA protocols may also be written as code in a programming language, most programming languages are not easily read by most researchers. Thus, the quality of scientific discourse on AA stands to gain from protocol descriptions that are easy to read, yet remain formal and readily executable by computers. This paper makes the case for using the HyperText Markup Language (HTML) to achieve this. While HTML can suitably describe AA materials, it cannot describe AA procedures. To resolve this, and taking away lessons from previous efforts with protocol implementations in a system called TEMPEST, we offer a set of custom HTML5 elements that help treat HTML documents as executable programs that can both render AA materials, and effect AA procedures on computational platforms.

## Introduction

Scientists in the social and medical sciences use intensive longitudinal methods (ILM) (Bolger & Laurenceau, 2013) for the repetitive sampling of individuals in the context of their daily lives and routines over extended periods of time. ILM are preferable over retrospective reports because the results are shown to be more valid for measuring actual experience, not having been compromised by memory biases (Moskowitz et al., 2009). Ambulatory assessment (AA) (Fahrenberg et al., 2007) is a type of ILM that employs several techniques, including the experience sampling method (ESM) (Csikszentmihalyi & Larson, 2014), the ecological momentary assessment method (EMA) (Stone & Shiffman, 1994), as well as monitoring environmental and physiological parameters through the use of electronic sensor devices, such as GPS, ambient light and noise sensors, or heart-rate monitors. The utilization of such technological elements makes smartphones very suitable for executing AA studies. As such, researchers conduct AA studies through software systems that help them implement and manage the study’s protocol.

A protocol is the plan for collecting data (Vogt & Johnson, 2011). In the context of a specific method, such as AA, the protocol includes two components: (1) detailed definitions of the *materials* (e.g., the instruments to be used), and (2) instructions on how the data collection *procedures* are to take place (e.g., detailed descriptions of how the instruments are deployed). There is agreement within the scientific community that the detailed representation of study protocols is important for research to be effective, replicable, and implementable (Michie et al., 2011), and calls are being made to share not only data but also materials and code (Nosek et al., 2015).

Yet, AA researchers emphasize the persistence of several issues in successfully reporting research that captures momentary assessments, contributing to a broader ‘replication crisis’ (or reproducibility crisis) in the social and medical sciences (Stone, 2017). Among those are issues of reporting on the data acquisition interface, and on the details of the sampling process (Stone & Shiffman, 2002), i.e., the materials and procedures of an AA protocol. As AA methods have become increasingly prevalent and accepted in the social and medical sciences over the last several decades (Riese, 2017), including broadly in the field of psychology, addressing the need to share AA protocols in a way that makes them easy to understand and implement at the same time is a noteworthy undertaking.

To describe AA protocols, researchers most often use natural language (as in Gunthert et al.,, 2007), and seldom other high-level representations, such as flowcharts (as in Ellis-Davies et al.,, 2012). In the present paper, we discuss why these practices make describing digital materials and procedures cumbersome, and their replication prone to errors.

Subsequently, we examine the software tools available to researchers for the implementation of AA studies, and show that they do not cater to the need for representing AA protocols to third parties in sufficient detail. To address this gap, we put forward a set of requirements for adequate AA protocol representations, which have to do with being both human readable and computer executable.

Finally, we propose a solution to satisfy the proposed requirements, one that takes advantage of the ubiquitous Hypertext Markup Language (HTML) and the Web browser. Importantly, while HTML documents are easy to read by both humans and machines, they cannot represent executable processes. Thus, we contribute a set of HTML5 custom elements that help treat an HTML document as an executable program. This document is representative of an AA protocol in both its materials and procedures, and serves as the single source for both the description and the implementation of an AA study. Using HTML documents is thus considered to reduce errors, and may foster easier replication of AA studies even when researchers may still use different systems to carry them out.

### Ambulatory assessment

As “a class of methods that makes use of mobile technology to understand people’s biopsychosocial processes in natural settings, in real-time, and on repeated occasions” (Conner & Mehl, 2015), AA methods share the motivations and include the procedures of ESM and EMA. In the remainder of this paper, as AA studies tend to collect both survey and sensor data, we will use the term AA as an umbrella term for various examples of smartphone-based ILM, including ESM and EMA. ESM, where participants are asked to regularly report their subjective experience, has traditionally focused only on survey data. Although EMA, where the interest of researchers extends also to physiological processes, has included sensor data, these have generally not been collected continuously. Examples of applications of AA methods can be found in the study of personality (Ebner-Priemer et al., 2007), mood disorders (Aan het Rot et al., 2012), substance abuse (Shiffman, 2009), or Parkinson’s disease (Hobert et al., 2014).

There is increasing interest in executing AA studies on smartphones, because they are relatively inexpensive and computationally powerful platforms. Smartphones support rich user interactions, e.g., through multi-touch screens. They can collect self-reports, as well as data about one’s physical context through embedded sensors, such as accelerometers, barometers, or ambient light sensors. Additionally, their capabilities can be augmented to continuously sample physiological data, through peripherals such as heart rate monitors. Moreover, study participants might already own the hardware, which makes it less costly to conduct a study.

In the digital domain, where AA studies with mobile devices take place, the *materials* used are instructive media such as text or images (representing surveys), graphical user interfaces (GUIs) for user input, sensors, and other hardware instruments such as clocks, data stores, or remote servers. The *procedures* that implement the studies are algorithms that operate on these materials, on the basis of longitudinal contingencies. As such, digital AA protocols are more intricate and complex than paper-based research protocols. Table 1 summarizes how procedures and materials in AA studies specifically differ from those in social or medical studies in general, due to the fact that the former depend upon digital means.

**Table 1:** Procedures and materials in AA studies reflect the fact that they are performed with digital means, as opposed to social and medical studies in general

	Social and medical studies	AA studies
Procedures	steps involved in completing the study from beginning to end, including:	steps involved in completing the AA component of the study from beginning to end, consisting of:
	∙ (chronological) details on participant recruitment	∙ methods for scheduling and initiating assessment sessions (e.g. timed triggers)
	∙ informed consent procedure	∙ conditional logic and order of instantiating computational processes
	∙ description of the test session(s)	
	∙ debriefing	
Materials	∙ paper-based questionnaires or other analog measures	∙ media on digital displays
	∙ computer programs as tools, interesting in terms of their inputs and outputs	∙ sensors that collect measurements
		∙ interfaces through which participants interact with the digital devices

## Issues with common representations of AA protocols

Typically, researchers describe research protocols in natural language, using regular prose. However, in the case of AA, the detail required for describing the study materials and procedures makes it difficult to use natural language to describe them, except only in high-level, abstract terms. For example, researchers will refer to the types of questions asked or sensors used, and will outline the sampling method, but will not go into depth with regard to the wording, or exact digital logic that initiates sampling sessions and processes data. A description in natural language is not concerned with how to implement a study protocol in software. As such, there may exist ambiguities at the lexical, syntactic, or semantic level, and as a result might allow the AA protocol to be converted into software in various ways (Ince et al., 2012). This can have negative consequences, such as a suboptimal presentation of the protocol’s materials, or a different order of the execution of the protocol’s procedures.

Besides the social and medical sciences, the problem with textual descriptions has been observed in other fields that use computational tools and methods to conduct research. Garijo et al. studied the reproducibility of an article, which described research in computational biology in textual terms (Garijo et al., 2013), and found that even though the authors had tried to make their work as clear as possible, eventually only experienced researchers managed to understand how to fully reproduce it Gil and Garijo (2017).

To escape the trappings of textual descriptions, professionals in software-related fields employ high-level notations, such as flowcharts (Nassi & Shneiderman, 1973), the Unified Modelling Language (UML) (Larman, 2001), process models (e.g., Petri nets Murata, 1989), or informal pseudo-programming languages (pseudocode) (Nishimura, 2003) to describe user interactions and processes. These notations, essentially combinations of mathematics and natural language, tend to be highly specialized to their professional domain, and are usually abstract, i.e., not concerned with specifics of implementation.

Although they have not been widely applied to AA protocols, an example of a flowchart for the description of a daily survey/questionnaire on motor development can be found in the continuous unified electronic (CUE) diary method (Ellis-Davies et al., 2012). An advantage of such a notation is that it provides a quick overview, and contributes to a better understanding of an AA study’s protocol. A disadvantage, however, is that researchers are burdened with the conversion of these high-level descriptions into usable implementations. In doing so, they also have to deal with possible ambiguities regarding the finer details that the description might not adequately cover. The conversion process requires significant effort and can be prone to mistakes, which, if detected at all, can render data unsuitable for the intended use, if the collection has already started.

Therefore, natural language is ambiguous, and high-level formal notations still leave the burden of implementation to the reader. To satisfy the demand that AA protocols be effective, replicable, and implementable (Michie et al., 2011), their representation has to cater to being understood by both its human users (i.e., AA researchers) and the computers it is executed on. Ideally, we wish for a singular representation that can be created, edited, and read by researchers with ease, but when inputted to computers can also incur the computational processes that conduct the study.

A representation that can be easily understood by humans is important for effective asynchronous collaboration within large-scale scientific communities, such as those in the social and medical sciences (Olsen et al., 1998). Olsen et al., (1998), however, warn of further limitations that computer systems can impose on the usefulness of representations of information. When it comes to handling such an information artifact in software, most digital tools support only the processing and transmission of data that match their own specific underlying data models (i.e., their decisions about how data should be structured and interconnected), and reject information that those data models do not support. The situation is exacerbated by incompatibilities between different software applications, and also within versions of the same application, which are issues that are often overlooked.

Olsen et al., (1998) proposed a number of requirements that might help make the process of creating, sharing, and eventually changing an information artifact (i.e., an item representative of a piece of information) more robust. For example, they noted that (1) the artifact must be communicated in a form that the collaborators can understand and manipulate; (2) all collaborators must possess technology that is compatible with the communication medium and that is readily usable for making changes to and comments on the artifact, and (3) the collaboration interface must be application independent, and must be under the control of message recipients, not message senders. At present, available configurations of AA systems are not concerned with providing representations of protocols that meet these requirements. The following section examines what software is available for managing and automating the data collection life-cycle, and why the AA protocol representations involved can be improved when it comes to issues of sharing and replication.

## Computers in ambulatory assessment

**Fig. 1:**
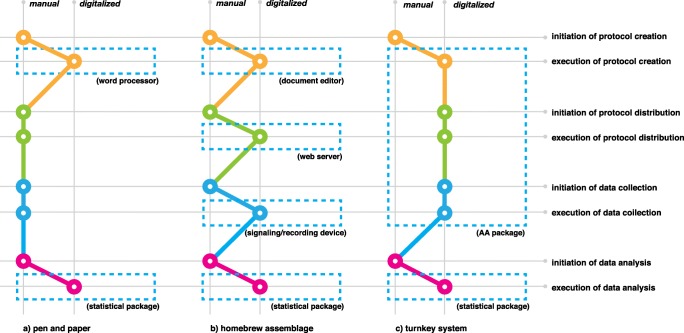
**a** Model of a paper-based data collection process in an ESM/EMA study. Though the preparation of materials and the analysis of data is done with computers (e.g., using word processors and statistical packages), all other data collection phases are initiated and executed manually. **b** ’Homebrew’ collection of discrete systems. Each part of the study can be performed with computers, but disconnected systems require manual intervention between output of one and input to another. Materials and procedures are inadvertently exposed, primarily as outputs and inputs to the various subsystems, and only implicitly can be taken as representative of the protocol itself. **c** An integrated (’turnkey’) system scenario. A large part of the process is handled internally but the materials are inscrutable and system-specific

The execution of an AA study can be broken down into phases, which include the preparation of the materials (protocol creation), their distribution to the participants (protocol distribution), collecting data (protocol execution), and analyzing data (Christensen et al., 2003). In terms of how each phase can be automated by computers, it is useful to pay attention not only to how substantially digital tools do support a particular phase, but also to whether the initiation of each phase can be automatically performed by computers, or demands the manual intervention of the researcher. Therefore, one can view the composition of software systems in support of AA studies as lying on a spectrum of options, where different compositions can be distinguished by how much of the process is performed either manually or in a computerized manner. Figure 1 models three such configuration instances. Each instance represents four phases of a study’s life-cycle in terms of both initiation and execution.

Figure 1a depicts a typical paper-based ESM/EMA study. It acknowledges that study materials are usually created on a computer, i.e., questionnaires are prepared in a word processor and printed out in large amounts on sheets of paper that would be handed to study participants to record data on. Opportunities for digitization exist at all other points.^1^

The rest of this section discusses two other exemplary configurations of AA systems, modeled in Fig. 1b and c. The first (homebrew assemblage) comes about by the combination of discrete tools that support each phase, but are independent of one-another. The second (turnkey system) is a tool that supports most of the AA study phases, by integrating corresponding tools into one package, and have them produce signals that denote that they have ended their work, or accept signals notifying them to start their work, thus increasing automation.

### Homebrew ambulatory assessment systems

One approach to using computers for AA studies is to assemble the system out of discrete, stand-alone subsystems, each dedicated to one task. These subsystems are largely agnostic of each other, and manual interventions are often required to initiate work. Figure 1b) models this approach. We use the term ’homebrew’ to describe this approach, which we derive from Voida and Harmon (2011). When studying the systems that non-profit organizations use to manage volunteers, Voida and Harmon (2011) found that most common information management systems are insufficient for the organizations’ needs. Volunteer coordinators resort to information management strategies that prominently utilize disparate pieces of off-the-shelf resources, media, and technologies. The authors characterized these assemblages of stand-alone software as ‘homebrew databases’.

In psychology journals, for example, one can readily find such stand-alone pieces of software, each dedicated to a specific task, corresponding to a particular phase of the AA protocol execution process. A homebrew AA system could be composed by picking, e.g., SurveyWiz (Birnbaum, 2000), a mobile short message service (SMS), and the Generic HTML Form Processor (Göritz & Birnbaum, 2005). SurveyWiz is a tool for producing HTML forms, the materials that participants would use to report data in a study. A SMS that includes a hyperlink can be used to signal participants to Internet-based questionnaires, so that signal-contingent data collection can take place. Such an example of a SMS-based setup can be found in Conner and Reid (2012). Then, the Generic HTML Form Processor can store the data submitted through it into a database for later processing. In the course of time, more general purpose software applications have also become prominent and common for producing surveys and storing the participants’ responses, as constituent components of homebrew AA systems, such as Qualtrics (Qualtrics, Provo, UT, USA) and Google Forms (Google, Mountain View, CA, USA), which are often used in conjunction with systems that help manage the complexity of administering notifications to study participants, like SurveySignal (Hofmann et al., 2015) (e.g., Righetti et al.,, 2016).

There are certain advantages of a homebrew approach to the composition of a data collection system. First, software components can be freely available to set up and to use, or may be obtained at low cost. Further, the inputs and outputs of the software for each phase are available to be inspected and modified. The fact that there needs to be manual intervention in order to move between stand-alone subsystems from one phase to the next affords researchers the flexibility to insert steps and components in-between as they see fit, to achieve goals in transforming materials or data.

However, the need for manual interventions can also be seen as a disadvantage, as manual operations in-between phases might be burdensome to conduct and can result in loss of automation, which software applications typically offer. Moreover, the resulting assemblage of systems can be daunting for non-experts to successfully manage. More often than not, these systems have not been specifically designed for interfacing with one-another, and getting them to co-operate demands the skills of an IT professional, such as operating Web and database servers, or having extensive knowledge of data formatting and data transformation practices.

The AA protocol representations used within these compositions of software necessarily adhere to the conventions used by their software components. They usually follow software-community standards, so in principle they can also be reused and fed into interchangeable software components. For example, the data that are produced by forms made with SurveyWiz could possibly be submitted to a program that accepts HTML form data, different from the HTML Form Processor. This makes the HTML forms, which SurveyWiz produces, representations of AA protocol materials that could potentially be shared with a wider community of researchers.

Although the sharing and reusing of such software artifacts (e.g., snippets of source code or scripts) is commonplace in communities that develop software, sharing of such digital materials has not commonly taken place with regard to AA protocols. For example, we could not find a repository where to inspect or copy e.g., the HTML forms used in a study. A possible explanation is that software-related representations, which express concerns of the corresponding generic software systems such as data formatting or client-server communications, are not bound semantically to the purposes of AA studies and might be considered too technical for researchers in the field. Understanding and manipulating the AA protocol when it is expressed in terms of computer software can therefore be hard and laborious for non-experts, requiring knowledge specific to software development.

### Turnkey ambulatory assessment systems

Discrete subsystems for each AA study phase can become interdependent, and merged into a larger system that can be thought of as a single software package specific to the configuration and execution of AA studies. Systems like the Electronic Mood Device (Hoeksma et al., 2000) or the Psymate (Myin-Germeys et al., 2011) in its form as a dedicated hardware device brought the preparation and distribution of materials and the data collection phases under one package. However, they still demand manual intervention from researchers to move from one phase to the next; researchers first used dedicated software to create the materials, then copy them as a configuration onto each device, then meet with each participant to hand the device over, and extract the data from each system after the end of the experiment.

As systems that serve different phases of an AA study become further integrated and interconnected through communications capabilities, they become more capable of initiating processes in a computerized, automated way, rather than a manual one. Figure 1c) models such a system found at the end of the manual-to-computerized spectrum. We refer to integrated systems as ‘turnkey‘, which is a term commonly used to characterize systems that have been “built, supplied, or installed complete and ready to operate” (Turnkey, n.d.).

Turnkey examples can be found in systems that are built for the purposes of a specific study and that implement its predefined protocol. Examples include Toss’n’ (Min et al., 2014), which was built for evaluations dedicated to sleep quality, and iHabit (Runyan et al., 2013), which targeted self-awareness. There are also more generic systems that allow diverse protocols to be configured, such as Ohmage (Ramanathan et al., 2012), Paco (Evans, 2016), the Aware Framework (Ferreira et al., 2015), Survalytics (O’Reilly-Shah & Mackey, 2016), Purple (Schueller et al., 2014), and the Experience Sampler (Thai & Page-Gould, 2017). Commercial applications also include LifeData (LifeData Corporation, Marion, IN, USA).

Illumivu (Ilumivu, Cambridge, MA, USA), Movisens (Movisens GmbH, Karlsruhe, Germany), and ESM Capture (ESM Capture, Cambridge, MA). Such packages offer graphical user interfaces (GUIs) with configuration options specific to the longitudinal nature of the data collection protocol. For example, configuring the manner of sampling over time, populating the survey items with adaptive text, setting the conditional presentation of specific survey items (referred to by some systems as ’skip logic’), or reusing the participant’s input at later points in time (referred to by some systems as ’pipping’).

Turnkey systems can make up for some of the disadvantages of homebrew collections in automating the data collection and in managing the entire study life-cycle. They also offer the advantage that researchers do not need to be concerned with implementation-specific and presentation-specific tasks, as the system developers have already made those choices, and they automate the generation of materials and the execution of the study. They do so by ensuring consistency of materials between the different processes, without requiring manual transformations to be performed in-between. They will, by design, make use of devices that participants already own as well as spare researchers the cost and burden of distributing hardware. Overall, current integrated systems make it easy for researchers to compose and modify a given protocol.

However, turnkey systems do not usually offer representations that depict the protocol concisely, in its entirety and in detail. Instead, the protocol is usually represented as a set of configuration options, distributed across several parts of the system’s GUI, and cannot be inspected or manipulated outside of that system. Also, there is generally no regard for compatibility between different systems; for example, there is no possibility to transfer an AA protocol implementation from Ohmage to Paco. To share their protocols, researchers would have to transcribe them manually to a different representation. Currently, that would usually be in natural language, which could possibly lead to weaknesses we have already discussed. In the next sections, we present an alternative, including a brief overview of the work from which it was developed.

## Motivation from past work

The work discussed in the present paper stems from our experiences in building, making available for use by researchers, and using TEMPEST (Batalas & Markopoulos, 2012b), a turnkey system for AA studies that helps build research protocols in a Web browser. The initial motivation for the system had been to provide a design wherein different concerns (e.g., database servers, user interfaces, textual contents) could be treated by different types of stakeholders (i.e., programmers, researchers), without them having to worry about implementation on levels other than their own (Batalas & Markopoulos, 2012a).

Researchers performing AA studies using this system were able to use configurable prepackaged components to piece their study materials together, specify what data they would be collecting as named variables, and set the sequences in, conditions under, and triggers after which the materials would appear to the participants in the study. By allowing sequential and conditional execution, and the writing and reading of variables, TEMPEST is unique in considering the study protocol itself to be an algorithm, running on a virtual computer, which is simulated by the software that participants use. The functionality that this virtual computer exposes is with regard to issues of immediate concern to the researcher, such as scheduling sampling sessions and choosing which interfaces participants should use for data input. Lower-level mechanisms, such as client-server communications, or the operations at the level of the smartphone’s operating system, are handled automatically.

TEMPEST has been deployed for the collection of data in 13 distinct real-world studies (Batalas et al., 2018), by different researchers, mostly in clinical psychology. It was perceived by the researchers to be a useful system that provided substantial benefits over paper-based methods or homebrew systems (see Fig. 1). The constructs for protocol composition that TEMPEST offers (configurable components, declaration of variables, sequential and conditional execution) proved effective in allowing researchers to carry out diverse studies as end-user developers of their own AA protocols.

These deployments also brought to light several opportunities for improvements. An important one was optimizing the ability to inspect the protocol’s details in succinct ways. Several studies involved multiple research assistants who helped with various aspects of the study’s execution, such as managing participants. Many were not the creators of the AA protocol, but rather maintainers or re-implementers. The AA protocol, as expressed in the GUI interface, did not make all of its details immediately apparent to these collaborators. For example, certain attributes were not visible in screen previews, but only during editing. Consequently, certain aspects of the protocol could escape a researcher’s attention, (e.g., that a variable name had already been used for a different survey item, or, when changing the name of an item, that items referring to it would also need to be updated) when reproducing a sequence of execution. Although not producing functionally erroneous behavior (protocol execution would not halt), this could lead to collecting different data from what was initially intended. We realized that it is important to provide researchers with representations of the protocol that are readable, unambiguous, and detailed enough so they can be easily shared, modified, and executed between different research teams and studies.

This observation led to the development of a new set of software components, distinct and independent from TEMPEST, which retain the ideas of using structures for sequential and conditional instantiation of protocol materials. This software also aims to satisfy the set of requirements for replicability, which we elaborate on in the next section.

## Requirements for AA protocol representations that foster replicability

We propose that the notation in which to compose AA protocols should meet the following requirements:

1. The document is human-readable and formally tractable, i.e., it is readable and editable by researchers, even without additional technological support, yet it can be transformed unambiguously through readily available computer programs (Khare & Rifkin, 1998).
2. The document can be executed, i.e., it should be possible for computer systems to perform processes in accordance with the AA protocol, without the need to produce separate or new artifacts.
3. The document is communicable and enables collaboration between different researchers (Olsen et al., 1998).
4. The document should be usable, i.e., specified users (researchers) achieve specified goals (conducting AA studies) in particular environments (institutions, academia) with effectiveness, efficiency, and satisfaction. This is in line with the cognitive dimensions framework (Blackwell et al., 2001), a set of principles for the design of notations, user interfaces, and programming languages, which proposes that the notation used in the document should facilitate the understanding of its contents, changes to its contents, and the creation of new content.
5. The document should be ensured to have sufficient support in terms of tools, i.e., software and hardware support are ubiquitously available in the present and in the future. It is often the case that system-specific representations go out of use once the software systems that produce and consume them become harder to access, e.g., because of obsolescence and lack of maintenance and support.

The contribution of the present paper lies in treating AA protocols as HTML documents, which meet all criteria listed above. We make use of HTML to create and deliver the protocol materials to the study participants. We achieve this by providing a set of custom Web components that make the declaration of materials easier, but also describe the manner in which the materials are used, i.e., how the protocol is to be executed, in the same document. Web browsers can formally parse the resulting document and drive the computational execution of the AA study protocol. The same document can be shared with other researchers to succinctly represent the protocol in all its detail, and to be adapted and modified for execution in other contexts.

The following two sections discuss why HTML is an appropriate candidate for satisfying the requirements stated, and how we can use modern HTML5 technologies to express the two aspects of a protocol: material and procedure.

### HTML for readable and executable AA protocols

Tim Berners-Lee introduced the World Wide Web in 1989, with the purpose of allowing researchers from around the world to share documents with each other. He also invented the Hyper-Text Markup Language (HTML) to overcome differences in how computer systems interpret the content of text files (e.g., how a line of text is demarcated) for display to the reader. As such, HTML provides a way to structure the content of digital documents (Berners-Lee et al., 1995). It is nowadays a ubiquitous medium for the transmission and presentation of information across different software platforms on devices with different form factors and intended functionality.

For the representation of AA protocols, one could consider other mark-up languages, besides HTML, that are used to structure documents in similar ways, by using tags. Tags are keywords, indicated to not be part of the actual text by being surrounded with the symbols <>. The programs that read these documents parse the tags with the understanding that they carry specific meanings. The Standard Generalized Markup Language (SGML) is an internationally agreed upon method of text mark-up that uses tags (Raggett, 1998). In fact, SGML gives HTML its notation of opening and closing tags. The eXtensible Markup Language (XML) (Bray et al., 1997) was introduced in 1996 as a subset of SGML. XML allows the application of the same principles to construct custom-made mark-up languages, which then require custom application programs to be interpreted by.

For the purposes of implementing AA protocols, we consider HTML to be a choice superior to other choices such as SGML and XML, primarily because of for the extensive support there is already in place for it in Web browser software. Web browsers are standardized pieces of software, available and up-to-date in all major operating systems, conform to the same set of standards, and are already familiar to end-users. They are also desirable for use for data collection, not only for their ability to render HTML documents, but also for other technologies they package, not least of which is their programmability.

Web browsers function by interpreting the mark-up structures in an HTML document and converting them into a graphically laid-out presentation of the document’s content. They are typically tasked with fetching an HTML document from a remote server and displaying it to the user, but also became programmable at a point early in their evolution. In more recent years, they have further evolved into powerful virtual machines, offering developers a fast, easy-to-program environment for general-purpose computing, which also acts as an intermediate between the programmer and the browser’s host device. The programmability of browsers has made possible the development of Web applications, software that dictates how the browser should interact with the user and what tasks are to be performed. The means by which Web-browsers are programmed is the JavaScript programming language. Thus, while HTML documents can instruct the browser with regard to the structure and presentation of information *materials* (e.g., text, images, video, or audio), programs for its JavaScript execution engine can specify the *procedures* to be performed by the device.

Normally, the specification of procedures to be performed comes at the cost of requiring considerable software development skills. In contrast, the effort required for the execution of AA studies with smartphones on the Web browser platform would become easier, given the ability to prescribe both materials and procedures in HTML, and not have to use JavaScript. HTML notation is formal and unambiguous in its semantics, easy for machines to understand, but still remains accessible to a wide human audience in terms of readability and ability to edit (Khare & Rifkin, 1998).

In the section that follows, we elaborate on our solution to the issue of describing both material and procedures in HTML. We use Custom Elements (Bidelman, 2007), a new technology in the HTML5 specification. We make use of Custom Elements in the context AA protocols for two reasons. The first reason is to allow our HTML scripts to be written using terms that relate to the concerns of the AA protocol, instead of using the syntactic elements of a typical Web page (see Tables 2 and 3). The second reason is to make HTML documents executable (see Table 4).

**Table 2:** A set of declarative custom elements to be used for the composition of AA protocols

aa-textfield: A textfield for free	<aa-textfield name="myText"	
text input.	label="allows text entry">	
	</aa-textfield>	
aa-boxgrid: A grid of boxes that lets	<aa-boxgrid name="myGrid"	
the user provide a two-dimensional rating.	vboxes= 5 hboxes= 5	
	width= 200 height= 200	
	top-label="top label"	
	bottom-label="bottom label"	
	left-label="left label"	
	right-label="right label">	
	</aa-boxgrid>	
aa-likert: Likert scale container,	<aa-likert name="myScale" five>	
essentially a specific configuration	</aa-likert>	
of aa-multiple-choice,		
with five or seven choices.		
aa-variable: Does not render an interface,	<aa-variable name="myVariable" value="myValue"></aa-variable>	
but sets a variable value in memory explicitly.		
aa-geolocation: Retrieves the device’s	<aa-geolocation name="myLocation"></aa-geolocation>	
GPS location.		
aa-function-random: Sets a variable	<aa-function-random name="myVariable" min="0" max="10">	
to a random value.	</aa-function-random>	

Some render interfaces for values to be provided by users and others produce values without user interaction. The names of these elements are semantically closer to AA protocol concerns than standard HTML element names such as < *div* >, < *span* >< *ul* >, < *li* >, for building protocol materials, or stand in as invocations of more complex functionality

**Table 3:** Custom Elements can accept and act on child elements, for greater freedom in configuration

aa-multiple-choice, aa-choice-item:	<aa-multiple-choice name="choices">	
A set of options to choose from, configurable	<aa-choice-item value="1">	
to allow either only one or many items to be chosen,	choice 1</aa-choice-item>	
and an item in a aa-multiple-choice set.	<aa-choice-item value="2">	
	choice 2</aa-choice-item>	
	<aa-choice-item value="3">	
	choice 3</aa-choice-item>	
	<aa-choice-item value="4">	
	choice 4</aa-choice-item>	
	</aa-multiple-choice>	
aa-screen: Groups contents together and provides	<aa-screen>	
a single “submit“ button for the user to signal	<div>Content can be placed as	
that this particular request for data was completed.	needed to communicate	
	with users</div>	
	<p><aa-textfield	
	label="please type something">	
	</aa-textfield></p>	
	<p>please make a selection:	
	<aa-likert></aa-likert></p>	
	</aa-screen>

**Table 4:** Procedural elements that specify logic

aa-choose, aa-when, aa-otherwise:	<aa-choose> .
Elements for conditional statements, modeled	<aa-when test="myVariable=='myValue'">'myValue' is the
after the corresponding XSL elements	value of myVariable</aa-when>
(Kay, 2017). aa-choose is the parent	<aa-when test="myVariable=='otherValue'">'otherValue'
container, the content of aa-when is instantiated	is the value of myVariable</aa-when>
when its condition is met, and the content	<aa-otherwise>myVariable has a different value</aa-otherwise>
of aa-otherwise is instantiated	</aa-choose>
when none of the conditions have been met.	
aa-sequence: Instantiates its children one	<aa-sequence>
at a time, as members of an ordered sequence.	<aa-screen>first</aa-screen>
Does so in response to received signals	<aa-screen>second</aa-screen>
(e.g., from the button press of an aa-screen).	<aa-screen>third</aa-screen>
	</aa-sequence>
aa-session: Container for the sampling	<aa-session
session, takes care of server communication	dates="01/08/2018;03/08/2018-05/08/2018"
and can be set to be triggered at specific	times="10:00;15:00;20:00">
times and dates.	</aa-session>

These procedural elements do not render interfaces, but instead determine how their children are to be instantiated

## AA protocol materials and procedures in HTML5

As already discussed, an AA protocol can be treated as having two aspects. The first aspect is concerned with the *materials*. This includes the text/image content that is presented to study participants, smartphones, sensors, or other features that are used, and addresses the ”what” of the protocol. In computer software terms, it is the *declarative* aspect of a protocol.

The second aspect is concerned with the *procedures*. This has to do with how the research materials are being put to use, e.g., their sequence order, or the conditions under which they are utilized. This aspect addresses the ”how” of the protocol. In computer software terms, it is the *procedural* aspect of a protocol.

Our goal here is to map both materials and procedures onto HTML elements, so that a single HTML document can act as both the description and the executable for an AA protocol (see requirements 1 and 2 as described previously). We are able to do this by defining custom elements, as allowed by the recent HTML5 specification.

Custom Elements (Bidelman, 2007) are part of a set of features specified by the World Wide Web Consortium (W3C), collectively known as Web Components (https://www.w3.org/standards/techs/components#w3c_all), which, when used in concert, enable users to define and distribute HTML elements that are not part of the W3C HTML standard. This allows us to define tags that are semantically close to a specific domain (in this case that of AA), and encapsulate within these tags programs with functionality for our own purposes (in the present case communication with servers and procedural execution). Tables 2 and 3 list some of the custom elements we have defined.

### Materials

**Fig. 2:**
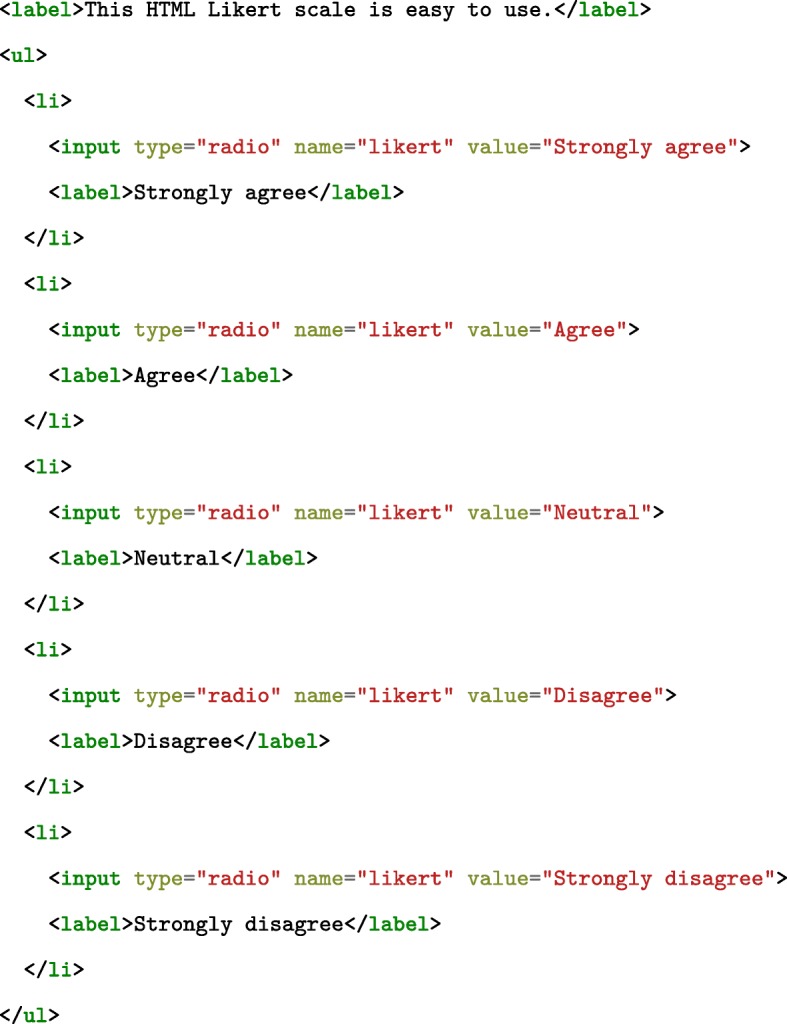
A Likert scale written in simple HTML. ul stands for unordered list, and li for list item. inputs of type="radio" under the same name="likert" represent a set of on/off buttons (radio buttons) where only a single one can be on at any given time. Note that extra code would be required to make use of the value the user reports (e.g., save it to a server)

The declarative aspect of an AA protocol, i.e., the *materials*, can be fairly straightforward to describe in standard HTML. Content can be structured as a regular Web page, including interactive elements that participants would be required to interact with. Figure 2 presents how a simple five-item Likert scale would be structured in HTML.

**Fig. 3:**
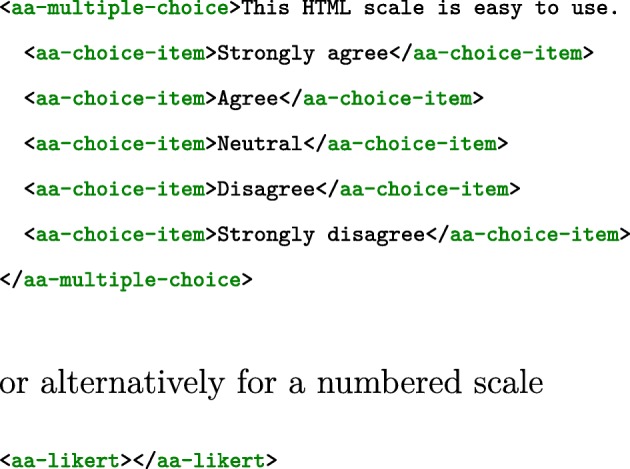
Likert scales written with our custom HTML5 components

Figure 3 presents the same five-item Likert scale written with our own custom Web component. With Custom Web Components, we provide a set of custom HTML elements that represent content commonly found in AA questionnaires, such as multiple-choice items, sliders, text-fields, with several configurable options. With custom HTML elements, the declaration of the instrument be in clear semantic terms, close to the task domain of AA, instead of the generic, purpose-agnostic syntactic terms of HTML (see Fig. 2).

Nardi (1993), in summarizing research on the learnability of formal languages by end-users, proposes that terms used in the language (language primitives) should map to concepts from the user’s domain. In this way, Nardi argues that users have an immediate understanding of what these primitives do (increased readability), can directly express their own operations as referred to in their own domain, and do not have to build them up using lower-level components (easier composition). In the examples of Figs. 2 and 3, our custom Web Components allow us to express a Likert scale for an AA protocol in the direct terms of aa-likert-scale and aa-likert-items, instead of the lower-level composition with an unordered list (ul), and its list items (li) of inputs and labels.

Additionally, custom elements can encapsulate functionality and eliminate concerns that are more specific to Web development than to the development of AA protocols. Potential Web development issues include browser compatibility, integration of different technologies (e.g., sensors), page layout, ensuring usability, user-interface design and implementation, and connectivity and synchronization with database servers (Rode et al., 2006). These can all be eliminated by way of delivering relevant functionality within the implementation of the corresponding custom Web Components, unlike the traditional Web development scenario, where such functionality of a Web page needs to be implemented alongside the creation of its content.

The collection of custom elements for AA can keep growing accordingly to accommodate other components of an AA protocol. Such elements do not have to include only simple user-interface widgets, but also widgets (i.e., reusable elements dedicated to a particular function of broader utility) that perform tasks in the background can be built. For example, a weather widget might capture information about the temperature at a particular location, to associate it with a response that the user provides at the same time. A random number generator widget could be used to help randomize certain instruments during a sampling session.

### Procedures

In contrast to the declarative aspect, the procedural aspect of a protocol cannot be defined using the standard set of HTML elements. Browsers render HTML documents in their entirety after loading them. Normally, the sequence of elements, as written one after another in a document, does not denote temporal order, but their spatial relations within the document’s flow as it could be read, from top to bottom. Within the scope of HTML, there is no way to specify that certain content should materialize at later times, or under certain conditions.^2^ Instead, to achieve such results, the JavaScript programming language is used to procedurally generate new HTML content and inject it into the document.^3^

In previous work, we presented how diverse protocols could be represented as programs in an environment that emulates a simple computer (Batalas et al., 2018): Interfaces and background functionality (*Widgets*) can be strung together in structures of *Sequential* execution. Also *Conditional* choose-when-otherwise structures can determine how the protocol progresses based on certain criteria. All these items operate on named variables stored in a memory structure. In addition to employing the *Widgets* as materials, with Custom Web Components we can provide the *Sequential* and *Conditional* structures as HTML elements, and allow the resulting HTML document to describe both what materials are used (declarative aspect), and how (procedural aspect).

**Fig. 4:**
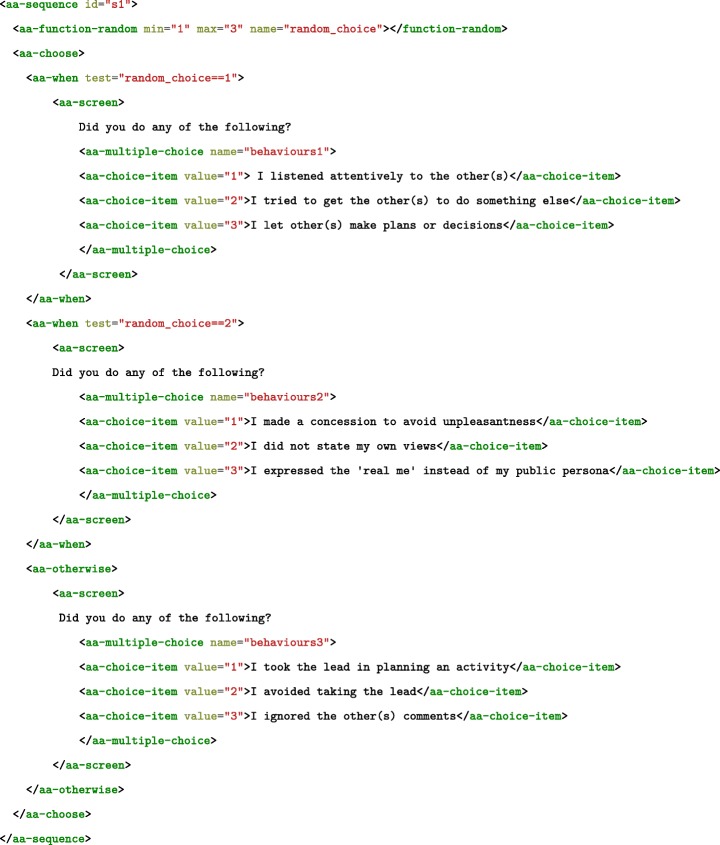
A parent sequence with two nested children. The first child is a random number generator storing values into the variable random_choice. After the first child finishes execution (i.e., generates and saves the value), the second child is instantiated. It is a choose-when-otherwise block that tests the variable random_choice for its value and conditionally instantiates one of three multiple choice groups, where the user is asked to indicate a behavior they have exhibited

In the case of *Sequences* (see Fig. 4), the parent <sequence> instantiates its first child element and waits for a control signal that signifies it is OK to move on to the next. In case of *Conditional* statements, the <choose> element is parent to a set of blocks of <when test="statement"> elements. The first <when> block with a statement that evaluates to true is instantiated. Optionally, an <otherwise> block specifies what happens when none of the test statements are true.^4^

Figure 4 provides an example of how sequence and conditional elements are used. Both make sure that their contents are ignored by the browser when the document is first loaded. The content is instantiated (i.e., is parsed by the browser, the code relevant to it is executed, and the results are committed to system memory) only when it is appropriate to do so. The fact that not everything is instantiated at the beginning as it happens with regular HTML content is important. It allows the protocol command of tasks (e.g., those that consume battery) only at the right time. One such example could be capturing a GPS position and phone orientation only at the time when a specific prompt is given to the user.

The script in Fig. 4 is a fragment of the event contingent recording (ECR) protocol described by Moskowitz and Sadikaj (2012), used by participants of an EMA study to describe and evaluate the social interactions of women with self-reported PMS (Bosman et al., 2018), and a study to evaluate the effects of alcohol consumption in a social context (Franzen et al., 2018). The full HTML ECR protocol can be found in the OSF repository by Albers et al., (2018), at https://osf.io/ftyh6/.

## Conclusion

Researchers who conduct AA (including ESM and EMA, or more broadly ILM) studies with computers and software systems agree that the field of psychology, or more broadly the social and medical sciences, can benefit from protocols that are not only effective and implementable, but also replicable. Computers solve several issues of automation, but also introduce stresses that pertain to expressing AA protocols concisely on the level of abstraction that is relevant to the field. An additional burden exists when having to implement a protocol description in any of the commonly used systems.

In this paper, we offered a definition of a study’s protocol as the information artifact that specifies both the study’s materials and the processes that use them. Materials provide information to participants and help capture their data, while processes control how materials are employed. We then proposed a cure to the issues of an AA protocol’s replication and implementation: Our proposition consists of using a single document that is both human readable and computer executable, and that represents both materials and processes.

Human readability is achieved by using HTML, a format for structuring digital documents that is widely understood by humans and supported by digital tools, now and probably also in the future. Readability for researchers in psychology is further enhanced by using custom elements to provide higher-level HTML syntax. These custom elements carry the benefits of shortening the syntactic verbosity of regular HTML, encapsulating complex functionality, and using names that semantically match instruments used in data collection. Computer execution is also achieved through a set of custom elements we built for the purpose of conducting AA studies. They allow researchers to control the flow of execution on the level that concerns serving participants with information, and capturing their input, and hide concerns that relate to inner-workings of the machine.

Such HTML-based AA protocols are easy to distribute through standard practices of the World Wide Web. It should also be noted that the approach does not reduce the expressive and programming power that HTML, JavaScript, and the browser’s runtime environment afford. The proposed components can be used in combination with other HTML structures and JavaScript code.

One issue to address is encouraging the scientific community to show interest in employing the approach proposed here, and overcome possible barriers, such as the following:

1. Although HTML is more accessible than code in most programming languages, its formal nature requires precision in using its syntax and following its conventions. This might be seen as a hindrance for certain users starting to read and write HTML text.
2. Additionally, it is easy to imagine certain protocols written now only using AA semantics-oriented custom elements, such as the ones proposed in Tables 2 and 3, but also mixed with the Web page syntactic elements of standard HTML, especially in cases where custom elements for a particular function do not exist or are inconvenient to write. This would result in landscape of fragmented protocol descriptions that vary in how close they are to the semantics of AA studies.
3. A third possible development would have different developers and vendors of AA systems creating and publishing their own brands of custom elements, so that e.g., company A might be encouraging the use of its own companyA-textfield and company B its roughly equivalent companyB-textbox. This might cause compatibility issues between different pieces of software and might call for the use of converters between their respective conventions.

Possible treatments for the above would be:

1. For assisting the editing of protocols, graphical user interface tools could guide authors to easily produce custom element configurations. Most turnkey systems already provide visual editing for their internal configuration, and could potentially support the proposed format as well. Also, it is possible to use AA protocol source text to produce visualizations that aid in its more immediate understanding.
2. In terms of battling fragmentation, aspects of protocols that find frequent use are more likely to eventually have custom elements written to represent them. Furthermore, repositories of protocols could help maintain and classify variations and versions of protocols according to aspects of their composition.
3. Societies in the field, such as the Society for Ambulatory Assessment, endorse the standardization of a basic set of components that are universally used in protocols, and provide guidelines for their behavior so makers of systems can provide corresponding implementations. Moreover, the availability of reference-component implementations will help towards standardizing the set of elements and their parameters, but also lower the barrier for application developers to integrate the functionality in their systems.

It should be noted that any specific software system is perishable, bound to be replaced by more newer pieces of software. More enduring, however, are ideas that software represents. While Web browsers have constantly been replaced or have evolved, HTML has endured, and grown as a standard. The HTML documents that browsers work with can function between multiple generations of software systems, gracefully shedding richer features when they are not supported. Similarly, an AA-executable document of the future might be different than what is proposed here. There is, however, the potential for the procedural execution in HTML, and the AA-related semantics that have been proposed here, which rely on features of HTML5 itself rather than any particular piece of software, to outlive specific implementations. As such, this work has laid out the possibilities that HTML and Web-related technologies can afford AA researchers and AA system developers in producing unambiguous, platform-independent, human-readable, and computer-executable representations of AA protocols.

## Footnotes

1 If the computerized initialization of a manual process might sound an oxymoron, consider the scenario of a digital wristwatch signaling a participant for input into a workbook at a given time (Delespaul et al., 2002).

2 A current practice in Web development that emulates the conditional appearance of content is to let the browser instantiate the declared elements as per its normal operation, but style them as non-visible, and toggle their visibility on certain events, e.g., mouse clicks.

3 JavaScript code is often distributed in separate files from HTML. It operates in a conceptual domain of object-metaphors and their properties, not the domain of the content that is served to the user, as HTML does, and requires a higher level of expertise to write than HTML.

4 The choose-when-otherwise elements are modeled after the elements of the same name found in XSL (Clark, 1999).
